# The strategies and outcomes of left subclavian artery revascularization during thoracic endovascular repair for type B aortic dissection

**DOI:** 10.1038/s41598-018-27588-7

**Published:** 2018-06-18

**Authors:** Yuwei Xiang, Bin Huang, Jichun Zhao, Hankui Hu, Ding Yuan, Yi Yang

**Affiliations:** Department of Vascular Surgery, West China Hospital, Sichuan University, Chengdu, China

## Abstract

This study was to analyze the outcomes of left subclavian artery (LSA) revascularization during thoracic endovascular repair (TEVAR) for type B aortic dissections (TBAD). From 2011 to 2017, TBAD patients who underwent LSA revascularization during TEVAR were enrolled. Technical success, endoleaks, mortality, complication, reintervention, and patency of target vessels were analyzed. 38 patients were included, 14 underwent carotid-subclavian bypass (CSB), and 24 underwent chimney graft (CG) implantation. Technical success rates were 92.9% and 100% in CSB and CG group. Eleven immediate type I endoleak (EL-I) was detected, including one from CSB group and ten from CG group. Three immediate type II endoleak (EL-II) was detected in CSB group. Perioperative complications showed no difference, but CSB group had longer intensive care unit (ICU) stay time. Median follow-up time was 26.2 months, and overall mortality was 14.3% and 0% in each group. Three EL-I and one EL-II underwent reintervention. All the LSA showed good patency, except one suffered from CG collapse. Both CSB and CG were feasible strategies to preserve the antegrade blood flow of LSA, and each strategy had its advantages and disadvantages. Based on our current experience, we preferred CG for high-risk patients. However, the evidence was still not strong enough, further well-designed studies are necessary to identify the criteria for LSA revascularization strategy during TEVAR.

## Introduction

For Stanford type B aortic dissection (TBAD), the thoracic endovascular aortic repair (TEVAR) had demonstrated favorable short- and mid-term results^1,2^. However, it had its limitations, one of which was the necessity of an adequate, disease-free proximal seal zones for the aortic stent graft. Anatomic challenges abounded for vascular surgeons because the lesions probably involving arch branches, usually left subclavian artery (LSA). A review of the literature suggested that 10–50% LSA needed to be covered intentionally to achieve an adequate seal zone^3^.

In 2009, Society for Vascular Surgery (SVS) published practice guidelines on the management of LSA during TEVAR, in which recommendations were made that the LSA should be revascularized before or after TEVAR as appropriate. However, the recommendations were based on low-quality evidence and did not address which revascularization strategy would be better during TEVAR for TBAD^4^.

In the present study, we shared our experiences in LSA revascularization by carotid-subclavian bypass (CSB) and chimney graft (CG), and analyzed the outcomes of these two strategies during TEVAR for TBAD.

## Materials and Methods

From January 2011 to April 2017, the data of TBAD patients who underwent TEVAR with LSA revascularization were reviewed. All the patients underwent preoperative CTA scans to evaluate the extension of aortic dissection, measure the distance between LSA and primary entrance, and determine the intended proximal landing zone (LZ).

At the initial stage, we preferred to CSB for LSA revascularization because of the sophisticated bypass technique. However, it was relatively traumatic and time-consuming. With the accumulation of experience, CG was preferred at the late stage.

All the TEVAR were performed under general anesthesia and with the use of intravenous heparin in a hybrid operation room or angiography suite. Valiant (Medtronic, USA) aortic stent grafts were used in all patients and proximally deployed in zone 2 (Z2), as specified by Ishimaru^5^. Except for emergent situations, CSBs were performed before the implantation of aortic stent grafts. The LSA was exposed through supraclavicular incision, PTFE vascular prostheses (GORE-TEX, USA) were anastomosed side-to-side between the second section of LSA and left carotid artery, and occluders (SHSMA, China) were implanted to close the orifice of LSA to prevent type II endoleak (EL-II). Self-expandable covered stent grafts (Fluency plus, Bard, USA) were used in the CG group. After the aortic stent graft was deployed, CG was deployed rapidly parallel to it with at least 1 cm overlapping and slightly protruding proximally.

Technical success, endoleaks, 30-day and overall mortality, perioperative complications, reintervention, and patency of target vessels were analyzed. Completion ascending aortic angiogram was performed to evaluate the immediate results. Technical success was defined as the instant postoperative aortic angiogram demonstrating successful implantation of the aortic stent graft, the lesion was excluded, and the LSA had favorable antegrade blood flow, no large amount of type I endoleak which needed to be converted to open surgery or implant another aortic stent graft proximally.

Follow-up examinations included CTA scan and ultrasonography, performed at postoperative 1, 3, 6, 12 months and then yearly thereafter. Symptoms and clinical signs were also collected during clinic visits.

SPSS 24.0 was used for statistical analysis. Continuous variables were expressed as mean ± standard deviation. The events were calculated with frequencies displayed as counts and percentages and analyzed by Fisher exact or χ^2^ probability test in a 2 × 2 table. A P-value of <0.05 was considered statistically significant. This retrospective study had followed the principle of the Declaration of Helsinki, the West China Hospital Ethics Committee had approved this study, and patient consent was waived.

## Results

A total of 38 patients (34 men, mean age of 54.1 ± 12.9 years, range from 31 to 84 years) were included, their characteristics were listed in Table 1. Among these 38 patients, 21 were in acute (within 14 days of onset) period, and 17 were in subacute/chronic (more than 14 days after onset) period. The indications of treatment were malperfusion syndromes in 3 patients, impending ruptures in 2 patients, aneurismal enlargement of false lumen in 8 patients, and failure of best medical management in 25 patients. Of the included patients, 33 had concomitant hypertension, 24 had smoking history, 8 had chronic obstructive pulmonary disease, 4 had coronary heart disease, and 3 had chronic renal failure. 14 CSBs and 24 CGs were performed for LSA revascularization, respectively. Elective settings were performed in 86.8% (33/38) and emergent in 13.2% (5/38) of all patients.

**Table 1:** Characteristics of patients.

	All (n = 38)	CSB (n = 14)	CG (n = 24)	P value
Age, year	54.1 ± 12.9	53.1 ± 12.9	54.8 ± 13.1	0.704
Men	34 (89.5%)	13 (92.9%)	21 (87.5%)	1.000
Acute	21 (55.3%)	9 (64.3%)	12 (50%)	0.393
Hypertension	33 (86.8%)	14 (100%)	19 (79.2%)	0.137
Systolic BP, mmHg	153 ± 29	157 ± 31	151 ± 29	0.564
Diastolic BP, mmHg	88 ± 16	90 ± 19	88 ± 15	0.693
Smoking	24 (63.2%)	8 (57.1%)	16 (66.7%)	0.557
COPD	8 (21.1%)	3 (21.4%)	5 (20.8%)	1.000
CHD	4 (10.5%)	2 (14.3%)	2 (8.3%)	0.616
CRF	3 (7.9%)	1 (7.1%)	2 (8.3%)	1.000
Emergency	5 (13.2%)	3 (21.4%)	2 (8.3%)	0.337
LCA involvement	5 (13.2%)	3 (21.4%)	2 (8.3%)	0.337

BP: blood pressure; COPD: chronic obstructive pulmonary disease; CHD: coronary heart disease; CRF: chronic renal failure; LCA: left carotid artery.

The technical success rates were 92.9% (13/14) and 100% (24/24) in CSB and CG group, respectively. One perioperative death occurred in CSB group due to severe acute renal injury. The length and diameter of proximal LZ were 17.9 ± 4.3 mm and 31.9 ± 4.2 mm respectively. A total of 51 aortic stent grafts were used. The diameter of aortic stent graft was 32.4 ± 3.9 mm (range 24–42 mm), the covered length was 215.8 ± 30.2 mm (range 150–280 mm). The diameter and length of CGs were 9.4 ± 1.1 mm (range 8–12 mm) and 56.7 ± 9.6 mm (range 40 to 80 mm). All the prostheses used in CSB were 6 mm in diameter.

Immediate EL-I was detected in 11 cases (11/38, 28.9%) on postoperative aortogram, including 1 from CSB group and 10 from CG group. These EL-Is were small amount, and did not need to be converted to open surgery or implant another aortic stent graft proximally. All of the CGs were balloon dilated at the same stage, 5 EL-I were mildly alleviated. Immediate EL-II was detected only in the three emergent patients from CSB group, who did not undergo LSA occlusion. Other perioperative complications included spinal cord ischemia (n = 2), pulmonary infection (n = 9), and incision complications (n = 5). The incidence rates of stroke and retrograde type A dissection were 0%. The hospital and intensive care unit (ICU) stays were 17.1 ± 7.6 days and 4.6 ± 5.9 days, respectively. Table 2 showed the perioperative characteristics.

**Table 2:** The perioperative characteristics of patients.

	All (n = 38)	CSB (n = 14)	CG (n = 24)	P value
Length of proximal LZ (mm)	17.9 ± 4.3	19.2 ± 4.7	17.2 ± 3.9	0.16
Diameter of proximal LZ (mm)	31.9 ± 4.2	33.5 ± 4.8	31.1 ± 3.6	0.08
Diameter of aortic stent graft (mm)	32.4 ± 3.9	33.9 ± 4.4	31.6 ± 3.4	0.08
Aortic covered length (mm)	215.8 ± 30.2	210.7 ± 26.2	218.8 ± 32.5	0.44
Mortality	1 (2.6%)	1 (7.1%)	0 (0%)	0.37
Immediate EL-I	11 (28.9%)	1 (7.1%)	10 (41.7%)	0.02^*^
Immediate EL-II	3 (8.1%)	3 (21.4%)	0 (0%)	0.04^*^
Spinal cord ischemia	2 (5.3%)	1 (7.1%)	1 (4.2%)	1.00
Pulmonary infection	9 (23.7%)	6 (42.9%)	3 (12.5%)	0.62
Incision complications	5 (13.2%)	1 (7.1%)	4 (16.7%)	0.63
Hospital stay (days)	17.1 ± 7.6	20.7 ± 10.2	14.9 ± 4.6	0.06
ICU stay (days)	4.6 ± 5.9	7.9 ± 8.4	2.7 ± 2.5	0.04^*^

LZ: landing zone; EL-I: type I endoleak; EL-II: type II endoleak; ICU: intensive care unit; ^*^P < 0.05.

**Figure 1:**
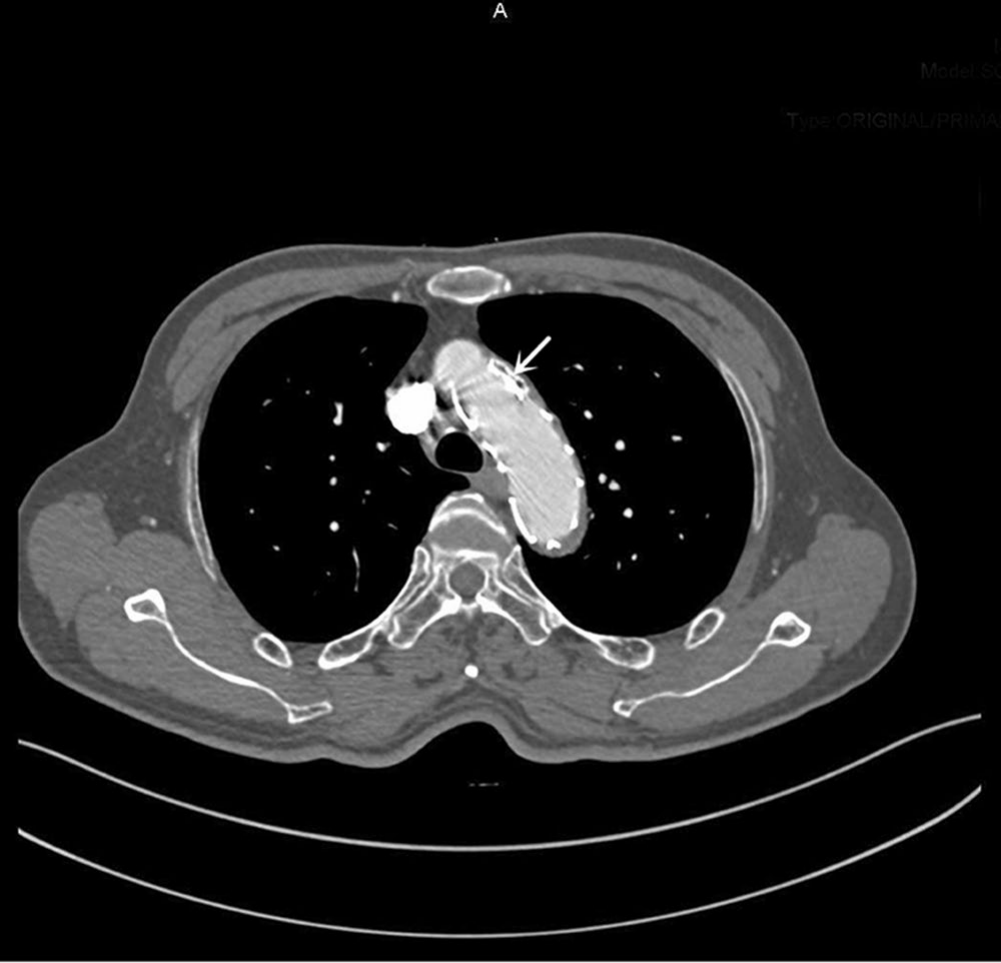
Chimney graft (white arrow) was compressed and occluded three months after the operation, without symptoms of ischemia.

**Figure 2:**
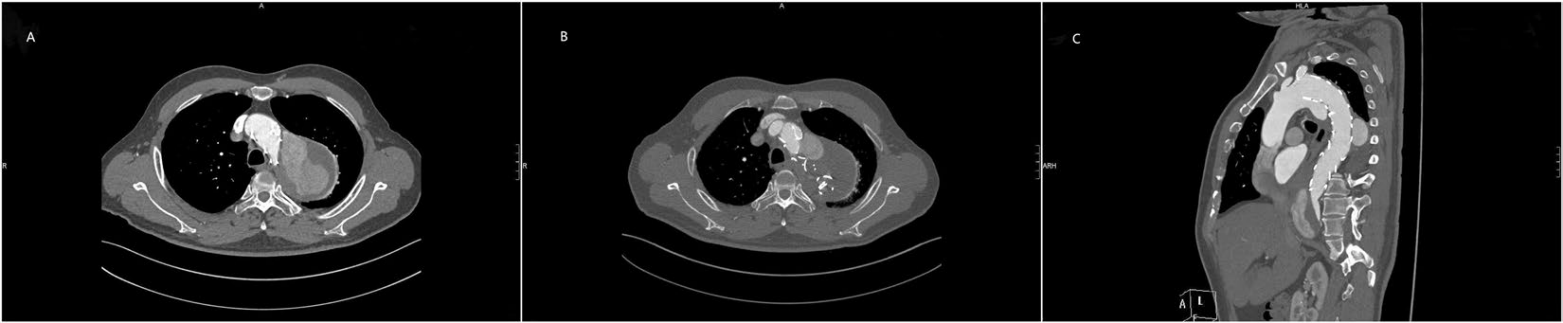
False lumen expansion caused by type I endoleak after chimney graft implantation (**A**); the type I endoleak was obviously alleviated after coil embolization of false lumen (**B**); aortic stent graft immigrant 20 months after the operation (**C**).

**Figure 3:**
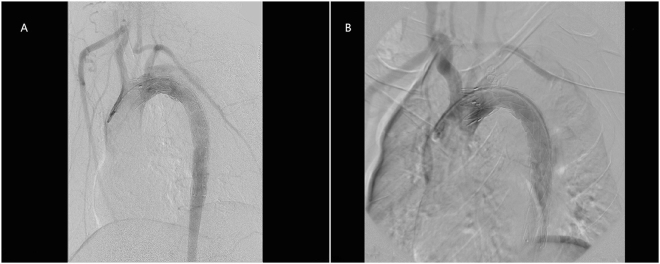
Type II endoleak after emergent carotid-subclavian bypass surgery in TEVAR (**A**); the alleviation of type II endoleak after coil embolization of LSA (**B**).

All of the 37 patients were successfully followed up with a median period of 26.2 months. During follow-up, one patient from CSB group died of respiratory failure two months after the operation. All the patients showed good LSA patency, except one suffered from CG collapse (Fig. 1). However, no reintervention was needed because ischemic symptoms were not presented. Among the 11 cases with immediate EL-I, 6 disappeared spontaneously (including 1 in CSB group and 5 in CG group), 2 underwent false lumen coil embolization because of false lumen enlargement, 1 underwent another TEVAR for aortic stent graft immigrant (Fig. 2). Others were stable without clinical consequences and followed up closely. Among the 3 cases with EL-II, 2 sealed spontaneously; another one received LSA coil embolization one months after operation (Fig. 3).

**Figure 4:**
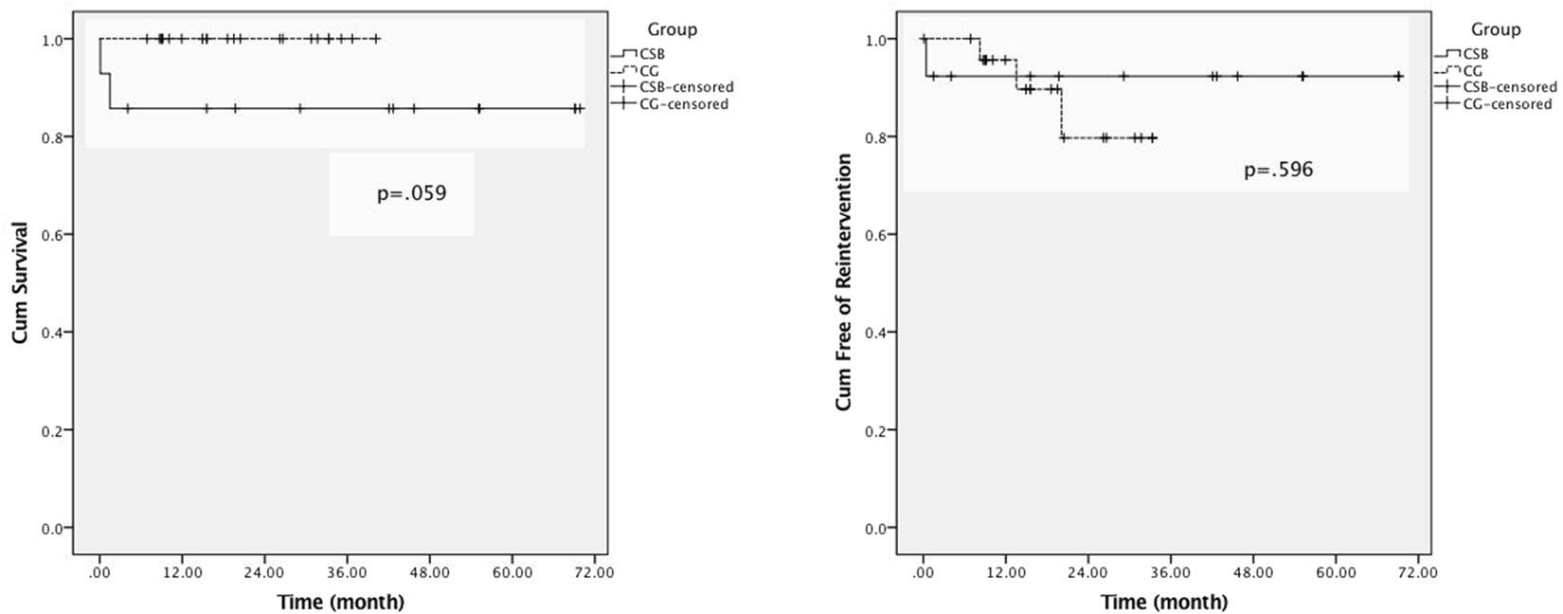
Kaplan-Meier analysis showed no significant difference in mortality and reintervention rate between groups. However, the events (death and reintervention) of CSB group were more likely to be concentrated in the early period of treatment and follow-up.

The outcomes during follow-up were listed in Table 3. Kaplan-Meier analysis (Fig. 4) showed no statistically significant difference in mortality and reintervention rate between groups. However, the events (death and reintervention) of CSB group happened earlier than CG group (1.04 ± 0.96 months vs. 13.95 ± 5.95 months, p = 0.02), and were more likely to be concentrated.

**Table 3:** Patients’ mid-term outcomes.

	All (n = 37)	CSB (n = 13)	CG (n = 24)	P value
Follow-up time (months)	27.9 ± 18.7	39.9 ± 24.1	21.3 ± 10.8	0.04^*^
Mortality	1 (2.7%)	1 (7.7%)	0 (0%)	0.35
Patency	36 (97.3%)	13 (100%)	23 (95.8%)	1.00
EL-I	5 (13.5%)	0 (0%)	5 (20.8%)	0.14
EL-II	1 (2.7%)	1 (7.7%)	0 (0%)	0.35
Reintervention	4 (10.8%)	1 (7.7%)	3 (12.5%)	1.00

EL-I: type I endoleak; EL-II: type II endoleak; ^*^P < 0.05.

## Discussion

TEVAR had shown favorable short- and mid-term result for patients with Stanford type B aortic dissection^1,2^, and LSA coverage was necessary to achieve good proximal fixation for those with an insufficient proximal landing zone. However, intentional coverage of LSA might not be physiologically tolerated because the LSA provides extensive circulation to the left upper limb, spinal cord and posterior cerebral circulation. A few of studies had reported their clinical outcomes on LSA revascularization during TEVAR, most of them only reported CSB or CG, no comparison had been made between these two strategies. We evaluated the available data regarding CSB and CG in TEVAR and summarized our experience with them.

Stroke is a major complication of LSA coverage in TEVAR. According to a meta-analysis on the morbidity and mortality during zone 2 TEVAR, LSA coverage was associated with an increased risk of stroke^6^. In the present study, no patient suffered from stroke perioperatively or during follow-up. Feezor *et al*.^3^ and Zamor *et al*.^7^ had reported that CSB could reduce the risk of stroke in TEVAR. And the reported stroke rate after CG implantation was lower than LSA coverage during TEVAR^8–11^. So both CSB and CG showed good effects on preventing stroke.

Spinal cord ischemia (SCI) is another devastating complication of LSA coverage in TEVAR^12^. Risk factors for SCI had been reported previously including the length of aortic coverage, prior abdominal aortic aneurysm repair, hypotension, and LSA coverage^13–15^. For patients with both extended aortic coverage and LSA coverage, LSA revascularization was necessary to maintain the perfusion of the spinal cord. In the present study, 18 cases had aortic coverage more than 240 mm in length, and 2 of them had postoperative neurological deficits (CSB n = 1, CG n = 1) referable to SCI. After cerebrospinal drainage and systemic blood pressure elevation, their symptoms were resolved. As reported^16^, hemodynamic control, cerebrospinal drainage, and neuroprotective drugs should be used during TEVAR for the patients associated with risk factors for SCI.

Patency was important for LSA revascularization. In the present study, all of the CSB prostheses and more than 95% CGs maintained patent during follow up. As reported by Martin *et al*.^17^, the patency rate of CSB in TEVAR was 95.2% (20/21) at a 74.6 months follow up. Similarly, Xue *et al*.^18^ reported that 91.1% (51/56) patients showed good CG patency during 16.5 months follow up. Besides, a multicenter retrospective study also reported a 98% primary patency of the CG^9^. So we believe both CSB and CG were effective method to maintain the antegrade blood flow of LSA.

Although it is rare, one CG was compressed and collapsed in our study, but no reintervention was needed. A few studies had reported the risk factors of CG compression. Some researchers had reported that CG compression was device related^19,20^, the radial support force was too strong in aortic stent graft or too weak in CG could both increase the risk of chimney stent compression or occlusion. Besides, in a study reported by Pecoraro *et al*.^21^, incomplete CG expansion, inadequate length and use in small and diseased target arteries were risk factors for occlusion.

Endoleak was another concern of LSA revascularization. The CG was expected to prevent endoleaks by prolonging the channel existed between CG and aortic stent graft. However, it was reported that the deployment of CG might potentially displace the aortic stent graft and induce an endoleak^22^. The immediate EL-I rate was significantly higher in CG group (7.1% vs. 41.7%, p = 0.02), and there were still five EL-I remained during follow-up, which led to reinterventions for false lumen enlargement and aortic stent graft immigrant. In a systematic review conducted in 2015^23^, the overall early EL-I rate of thoracic CGs was 11%, and 42% ELs-I were treated by embolization, extension of aortic stent graft or even open conversion. Recent studies^24–26^ also reported a similar result. So we believed that CG brought more EL-I and had a higher chance of reintervention.

Concerning the indication of CSB and CG in TEVAR, they can be both applied in the cases requiring LSA revascularization, especially for those with dominant left vertebral artery or left upper extremity dialysis access. However, no criteria had been reported that which method should be chosen for LSA revascularization. There was no doubt that CSB was more traumatic than CG, which could result in longer operation time and more complicated postoperative course. In the present study, the ICU stay time was significantly longer in CSB group than CG group, and the pulmonary infection rate was higher in the bypass group (42.9% vs. 12.5%). Besides, the events of CSB group concentrated in the earlier than CG group, so we preferred CG for high-risk patients (comorbidities, advanced age or low cardiac function) for a stable postoperative course. Otherwise, CSB was recommended for low-risk patients who can suffer a relatively comprehensive perioperative course, or who cannot be followed up for a long time since the events barely happened in the late stage of follow up.

The key strengths of our study were that TBAD was the only indication for TEVAR and the characters of both groups were similar. However, the limitation of our study was the small sample, and the different follow-up time between groups (37.1 ± 25.5 months vs. 21.3 ± 10.8 months, p = 0.043), so the long-term result could be different as the follow up continued. Besides, due to non-randomization and a retrospective design, the study is subject to inherent bias and confounding. Further controlled studies are necessary to identify the first choice of LSA revascularization strategy for TBAD patients.
